# Changes in Lipid Profiles of HIV^+^ Adults over Nine Months at a Harare HIV Clinic: A Longitudinal Study

**DOI:** 10.1155/2016/3204818

**Published:** 2016-03-08

**Authors:** Danai Tavonga Zhou, Doreen Nehumba, Olav Oktedalen, Princess Marange, Vitaris Kodogo, Zvenyika Alfred Gomo, Tonya M. Esterhuizen, Babill Stray-Pedersen

**Affiliations:** ^1^Institute of Clinical Medicine, University in Oslo, Oslo University Hospital, P.O. Box 1171, Blindern, 0318 Oslo, Norway; ^2^Department of Medical Laboratory Sciences, College of Health Sciences, University of Zimbabwe, P.O. Box AV 178, Avondale, Harare, Zimbabwe; ^3^Division of Community Health, Faculty of Medicine and Health Sciences, Stellenbosch University, Francie van Zijl Drive, Tygerberg, Cape Town 7505, South Africa; ^4^Department of Infectious Diseases, Oslo University Hospital, P.O. Box 4950, Nydalen, 0424 Oslo, Norway; ^5^Department of Chemical Pathology, College of Health Sciences, University of Zimbabwe, P.O. Box AV 178, Avondale, Harare, Zimbabwe; ^6^Centre for Evidence-Based Health Care, Faculty of Medicine and Health Sciences, Stellenbosch University, Francie van Zijl Drive, Tygerberg, Cape Town 7505, South Africa

## Abstract

HIV infection, together with ART, is associated with changes in biochemical, metabolic parameters and lipid profiles. The aim of this study was to compare changes in lipid profiles among HIV positive outpatients over nine months. 171 patients were investigated, 79% were ART experienced, and 82% of ART experienced patients were on NVP/EFV first line at baseline, but some patients changed ART groups over follow-up and classification was based on intent to treat. More than 60% ART naïve and ART experienced patients had some form of dyslipidemia either at baseline or at follow-up, but mean lipid values for the two groups were within normal limits. At baseline and follow-up, mean levels of TC and HDL were slightly higher in the ART experienced group. Interestingly, there was higher increase in HDL over time in the ART negative group compared to the ART positive group. There was a decrease in TC/HDL ratio in both groups over time, suggesting a reduction in calculated risk of CHD over time. HIV positive patients frequently show various forms of dyslipidemia, but there are no changes in average atherogenic lipid levels and results suggest reduced risk of CHD, mainly due to increases in HDL, after nine months of observation time.

## 1. Introduction

To date, HIV remains a major public health threat in Africa as a whole and in Zimbabwe alone where prevalence of HIV-infected adults is 14.4% [1, 2]. Though HIV infection and antiretroviral therapy (ART) are often associated with a variety of changes in biochemical and metabolic parameters including changes in lipid profiles, ART regimens have revolutionized the care and management of acquired immune deficiency syndrome (AIDS) due to HIV and have transformed the disease from a life-threatening infection into a chronic and manageable condition [1, 3].

Whilst ART does not cure AIDS and is therefore taken for life, it reduces morbidity and mortality, if used appropriately [1, 3]. The national ART program in Zimbabwe began in April 2004, and since that time the benefits of such therapy have been widely documented in the country [4]. Current World Health Organization and 2013 antiretroviral guidelines for Zimbabwe recommend a preferred first-line regimen for adults, adolescents, and older children of two nucleotide/nucleoside reverse transcriptase inhibitors (NRTIs), for example, tenofovir (TDF) and lamivudine (3TC) together with nonnucleotide/nucleoside reverse transcriptase inhibitor (NNRTI), for example, efavirenz (EFV) and nevirapine (NVP) and a second-line ART regimen of boosted protease inhibitor (PI) supported by NRTIs [4–6].

Infection with HIV impairs the reverse cholesterol transport (RCT) process in macrophages and monocytes; hence clinical observations have documented dyslipidemia in patients with AIDS and symptomatic HIV infection. The earliest alterations recognized, in terms of disease stage, are decreases in high density lipoprotein (HDL) and low density lipoprotein (LDL) concentrations [7]. Further clinical observations, results of clinical trials, and results of studies in healthy adult volunteers have documented metabolic effects on lipid metabolism by NRTIs, NNRTIs, and PIs [8, 9]. NRTIs are associated with alterations in body fat deposition and metabolic alterations, due to drug accumulation within adipocytes resulting in mitochondrial dysfunction [10]. On the other hand, NNRTIs such as efavirenz (EFV) and nevirapine (NVP) [9, 11] have been associated with favourable lipid profiles. In particular, ART regimens containing NVP are associated with a better lipid profile, mainly because they provide higher serum concentrations of HDL [12]. Patients who use PIs for a long period of time, however, frequently present with hypertriglyceridemia, elevated concentrations of LDL, and reduced HDL levels, all of which are atherogenic changes [13–15].

In the 987 Home Based Aids Care Program [16] a study of 374 patients was carried out in Uganda; mean serum lipid concentrations of TC, LDL, and HDL increased after 24 months of NVP/EFV treatment, in agreement with an Indian study showing an increase in TC and TG levels after 20 months on NVP/EFV regimens. Furthermore, a multicenter study from Madrid showed an increase in TC after 12-month treatment with PI-based drug regimen comprising lopinavir and ritonavir (LPV/RTV) [16–18].

Clinicians offering health care to HIV positive patients need to be aware of the various clinical and biochemical presentations and hence to keep a high level of suspicion. Due to the presence of contradicting study results, it is not clear where the Zimbabwean population stands with regard to changes in lipid profiles in patients on different ART regimens, over time. In the current era of HIV infection and ART, knowing patients' risk and acting to reduce it are imperative to long-term survival. The aim of this longitudinal study was to determine and to compare the changes in lipid profiles in ART experienced and ART naïve patients previously described at baseline [19] after nine months of follow-up.

## 2. Materials and Methods

### 2.1. Study Design, Study Site, and Recruitment

This was a cohort prospective observational study. Documented HIV-infected patients aged 18 years and above who attended the HIV treatment clinic in Harare between March and August 2013 were consecutively recruited into the study after giving informed consent [19] and followed up nine months later. Demographic and clinical data were as follows: age, sex, marital status, health status, clinical history, and family history were collected at baseline [19]. Blood samples in plain tubes were collected at baseline and nine months later, separated and stored at −80°C, and then thawed once before analysis.

### 2.2. Ethical Considerations

Ethical clearance to carry out the study was granted by the Joint Research Ethics Committee of the University of Zimbabwe College of Health Sciences and Parirenyatwa Group of Hospitals (JREC) (173/11), Medical Research Council of Zimbabwe (MRCZ) (MRCZ/B/352), and Norwegian Research Ethics Committee (REK) (20121100). Permission to access patient samples and data was granted by the clinic research committee after they were satisfied that the research protocol would not interfere with the usual clinic practice.

### 2.3. Quantitative Determination of Lipids

TC, HDL, and LDL were measured enzymatically in serum in a series of coupled reactions as described earlier [19].

### 2.4. Statistical Analysis

Stata® version 13 (StataCorp, Texas) was used to analyse the data. A *P* value < 0.05 was considered as statistically significant. Baseline continuous, normally distributed variables were compared between the two independent groups using *t*-tests, and categorical variables were compared using Pearson's chi square tests while binominal results at the two time points were compared by McNemar's chi square tests using the exact binomial probabilities. Generalised linear models for the change in outcome values over time were constructed for each lipid (cholesterol, HDL, LDL, and TC/HDL ratio) whilst independent variables tested included ART history, sex, age, and BMI at baseline. The models used robust standard errors to adjust for the clustering in repeated measures of patients at two time points. The interaction between time and ART history was tested, and, if statistically significant, remained in the model and was interpreted rather than the main effects of time and ART history. Dyslipidemia was determined as TC > 5.2 mmol/L, HDL < 1.1 mmol/L, LDL > 3.2 mmol/L, and TC/HDL ratio > 4.5, according to National Cholesterol Education Program, Adult Treatment Panel III (NCEP ATPIII) guidelines [20].

## 3. Results and Analysis

### 3.1. Demographics of Participants

215 HIV-infected adults were included in the study at baseline [19], 171 (79%) were accessed at follow-up, four (2%) had died due to AIDS-defining illnesses before follow-up, six (3%) transferred from the clinic, 25 (12%) were lost to follow-up at the clinic for unknown reasons, and nine (4%) had incomplete follow-up data. Patients who were eventually declared as lost to follow-up for unknown reasons were aware that they were required to attend the study for a second visit and when tracked by telephone, they declined most likely due to fear of stigma associated with HIV in many populations of the world [6] or economic reasons associated with low income in this study population [19].

There was no difference in sex, marital status, employment status, mean earnings, and mean body mass index (BMI) between the ART naïve and ART experienced patients at baseline and follow-up (Table 1). However, based on baseline data, ART experienced patients were generally older than the ART naïve patients and the majority of the participants were women [19]. Most of the patients were on first-line ART comprising mainly TDF/NVP/3TC and TDF/EFV/3TC while very few (7.4%) were on PI-based second-line regimen, at baseline (Table 1). 20% of ART^+^ patients had switched drugs to either alternative first-line or second-line drugs and 8% of ART^−^ patients had started ART before second visit. More ART experienced patients than ART naïve patients, classified using intent-to-treat, were on treatment for hypertension, both at baseline [19] and at follow-up nine months later.

By comparing baseline and follow-up data, average TC and HDL levels were significantly higher in ART experienced patients than in ART naïve patients while there was no difference in the mean LDL levels. Specifically, mean TC level of ART experienced patients was on average 1.44 mmol/L higher than for those who were ART naïve, at follow-up (*P* < 0.001), after controlling for sex, age, and baseline BMI as confounders (Table 2). However, those on ART showed a highly significant rate of decrease in TC over time compared with those who were ART naïve (*P* = 0.001, Table 2).

At follow-up ART experienced adults, the majority of whom were on NVP/EFV first line, had an average HDL level which was 0.603 mmol/L higher than those who were ART naïve (*P* < 0.001) after controlling for baseline diastolic blood pressure and BMI. Those who were ART naïve increased their HDL values over time at a significantly higher rate than those who were on ART (*P* = 0.002).

Also at follow-up patients on ART had LDL level which was on average 0.146 mmol/L higher than those who were ART naïve, but the difference was not statistically significant (*P* = 0.405) after controlling for baseline systolic blood pressure. There was no significant interaction between time and group, but there was an increase in the LDL values in the ART naïve patients while a decrease in the ART experienced patients was observed.

Of note, there was an overall decrease in TC/HDL ratio in both groups over time, which was, however, not different between the groups. There was no overall difference between the groups in terms of this outcome (0.841) after adjustment for age and baseline BMI (Table 3).

Nineteen out of twenty-three (83%) of ART naïve patients compared to 79% (*n* = 117) of ART experienced patients (*P* = 0.694) had some form of dyslipidemia, at baseline (Table 4), when classified by absence or presence of any one of the characteristics of the National Cholesterol Education Programme Adult Treatment Panel III [20] for TC, LDL, HDL, and TC/HDL ratio. At follow-up, there was still no difference in frequency of dyslipidemia when the two groups were compared (61% against 65%, *P* > 0.05). By comparing data as matched pairs at baseline and nine months, respectively, there was no significant difference in the proportions of patients with overall dyslipidemia over nine months for ART naïve patients, *P* = 0.064, but a significant drop in the frequency of ART experienced patients with dyslipidemia (−14.2%, *P* < 0.001) (Table 5).

The prevalence of ART naïve patients with elevated TC (>6.21 mmol/L) showed a slight decrease from one patient to none (Tables 4 and 5), and the proportion of ART naïve patients with elevated LDL and depressed HDL (>1.1 mmol/L) showed slight decreases which did not reach statistical significance (Table 5). A similar pattern was observed for ART^+^ patients: there were slight decreases in prevalence of hypercholesterolemia as measured by TC, 37.2% (*n* = 55) at baseline versus 15.9% (*n* = 23) at follow-up, *P* = 0.281. For ART positive patients, proportion of patients with depressed HDL and elevated LDL however decreased significantly over the nine months of follow-up, *P* < 0.0001 (Table 5). The elevated TC/HDL ratios, a measure of coronary heart disease risk, showed a decrease over time in both patient groups (Tables 4 and 5).

## 4. Discussion

In this HIV population study the serum levels of TC, HDL, LDL, and calculated TC/HDL ratio in both ART naïve and ART experienced patients were all within physiological levels (Table 1). However, many of the patients had some form of dyslipidemia which implies an increased risk to the development of coronary heart disease in these patients. At baseline 83% of ART naïve and 79% of ART experienced patients had evidence of some form of dyslipidemia and the high prevalence remained at follow-up nine months later.

Comparing the lipid levels by ART experience showed a significant difference in serum TC and HDL at baseline [19] and follow-up (Table 2). Average serum TC and HDL concentrations were significantly higher in the treated group at baseline and at follow-up nine months later, while there was no difference in LDL and TC/HDL ratios between the two groups (Table 2). Interestingly, TC decreased over time while HDL increased over time; the ART positive patients showed more rapid decrease in TC and less rapid increase in HDL than the ART negative patients. The higher levels of TC in treated patients are worrying because prolonged elevated levels of TC (and LDL) increase the development of atherosclerosis [21].

Our finding is partly consistent with the findings from the Multicenter AIDS Cohort Study (MACS), a multicenter prospective cohort study of men in four locations of the USA. In the MACS study ART initiation was associated with increases in TC, LDL, non-HDL, and TC/HDL ratio. The atherogenic lipid profiles occurred shortly after ART initiation and lent support to the recommendation for baseline and serial lipid measurements as a standard of care in the management of HIV treatment [22]. Of note is that at baseline in our longitudinal study the ART positive patients had already been on their combined antiretroviral treatment for mean of 3.5 years [19] while the ART negative patients were still naïve of antiretroviral treatment, at study baseline. Although the degree to which serum lipid abnormalities contribute to the risk of cardiovascular events in HIV-infected persons is not well established, low HDL level in an untreated patient is of particular concern as this lipid abnormality is least amenable to pharmacological therapy. Encouragingly, in our study the average HDL level increased in both groups at follow-up, in contrast to reports from the MACS study, which reported persistence of reduced HDL level after ART [22]. HDL increased more rapidly in the ART negative group, in our study, which is surprising as we expected steeper increase in the ART positive group. Could HDL increases in ART naïve patients be due to improved medical care and dietary improvements as the clinic offers supplements to patients from disadvantaged backgrounds? This requires further enquiry, bearing in mind that low HDL levels have previously been associated with longer duration of HIV infection and higher levels of HIV RNA in circulation [10] and we have previously reported that the duration of HIV infection was longer in ART positive patients [19]. It is however difficult to make definite conclusions as data on viral loads and CD4 counts was not available.

When using NCEP guidelines for defining dyslipidemia there were some interesting findings when comparing the two patient groups over time. There was a reduction in prevalence of ART positive patients with depressed HDL and elevated LDL over the nine months of follow-up, whilst proportions of ART naïve patients with depressed HDL, elevated LDL, and elevated TC remained the same. This is suggestive of decreased prevalence of high risk of coronary heart disease in ART positive group due to HDL recovery and unchanged prevalence of high risk in ART naïve patients over time, considering that high risk is associated with elevated TC and low HDL in the general population, over time [20].

In the study of rural Ugandans with advanced HIV disease initiating NVP- or EFV-based ART there were infrequent elevations in TC, LDL, and TG at baseline and after 24 months of therapy [23]. Increases in HDL levels were substantial and proportionally greater than increases in TC or LDL levels. The risk of coronary heart disease and how it was affected by lipid changes in this rural African population was not investigated but is expected to be low. Any differences between these findings and those of the current study could be due to variations in race/ethnicity, dietary, environmental, lifestyle factors, and different study designs [24, 25].

In agreement with our study results, an earlier Zimbabwean longitudinal study on DART patients over forty-eight weeks reported low levels of TC, LDL, and TGs at baseline (time of switch to second-line, after 2.2 years on first-line ART). After approximately forty-eight weeks of second-line ART, however, patients were reported to have marked increases in lipid levels, although TC/HDL ratios remained unchanged. Higher proportions of patients at follow-up compared to proportions at baseline had TC and LDL levels that were greater than normal, whereas lower proportions of patients had depressed HDL, *P* < 0.001. There was no difference in proportions of patients with elevated TG and elevated TC/HDL, *P* > 0.15, suggesting no increase in prevalent risk of coronary heart disease over the forty-eight weeks of follow-up [26]. In a South African longitudinal study TG and cholesterol levels increased significantly in patients on stavudine-based first line [27]. Stavudine is however rarely used in our study clinic so comparison of our results with this South African study is limited.

## 5. Conclusion

In conclusion, this present study does confirm that HIV positive patients, either ART negative or ART positive on NVP/EFV first-line regimen, show dyslipidemia, although some changes over time are beneficial. There is still a need of monitoring the lipid levels routinely in HIV patients who are either ART naïve or on first-line ART considering that the Zimbabwean HIV-infected population is getting older and steadily increasing its risk of coronary heart disease.

## 6. Limitations

Although the present study has reported relationships between HIV positivity, ART exposure, and dyslipidemia, the observational nature of the present study prevents an establishment of causal relationships between the HIV infection, the various ART drug regimens, dyslipidemia, and final coronary heart disease. The study could also not provide evidence for changes in lipid and coronary heart disease risk according to type of ART, mainly due to small sample size of the groups on EFV-based first line and PI-based second line as small sample sizes lead to imprecise estimations.

## 7. Recommendations

The study size needs to be larger to be more representative of the Zimbabwean population, improve power of study, and reduce type II errors, so as to confirm the findings in this study. A longer longitudinal study on a larger population that takes note of both viral loads and CD4 counts, together with the clinical and biochemical measures, will provide stronger evidence linking HIV and ART to dyslipidemia and coronary heart disease. This evidence is urgent in Zimbabwe as the HIV-infected population not only survives longer, but also is rapidly aging and hence is more at risk of coronary heart disease.

## Figures and Tables

**Table 1:** Demographics of participants (*N* = 171).

Variable	ART^−^ (*n* = 23)	ART^+^ (*n* = 148)	Total	*P*
Age/years				
Mean ± SD	36.4 ± 11.2	41.2 ± 10.2	40.5 ± 10.4	0.0417^*∗*^

Sex				
Male/*n* (%)	6 (17.1)	29 (82.9)	35 (20.5)	0.473^*∗∗*^
Female/*n* (%)	17 (12.5)	119 (87.5)	136 (79.5)	

ART use at baseline	23	148	171	
NVP/EFV first line	—	126 (85.2%)		
ZDV/STV first line	—	11 (7.4%)		
PI-based second line	—	11 (7.4%)		

Earnings in USD mean ± SD	126.0 ± 143.3	147.8 ± 190.2	144.9 ± 184.4	0.5995^*∗*^

BMI mean ± SD	23.5 ± 4.0	24.8 ± 4.9	24.6 ± 4.8	0.2344^*∗*^

SBP mean ± SD	125.0 ± 18.8	125.9 ± 18.9	125.8 ± 18.8	0.8154^*∗*^

DBP mean ± SD	81.9 ± 18.8	81.7 ± 15.0	81.7 ± 15.5	0.9558^*∗*^

Note: level of significance is set at *P* < 0.05; ^*∗*^ *t*-test comparison of means; ^*∗∗*^Pearson chi-squared test; SD: standard deviation; BMI: body mass index; SBP: systolic blood pressure in mmHg; DBP: diastolic blood pressure in mmHg; ART^−^: antiretroviral therapy naïve at baseline; ART^+^: antiretroviral therapy experienced at baseline; NVP: nevirapine; EFV: efavirenz; ZDV: zidovudine; STV: stavudine; PI: protease inhibitor.

**Table 2:** Generalised linear model for total cholesterol.

	Coefficient	Robust standard error	*P*	95% confidence interval	
				Lower	Upper
Follow-up versus baseline	−0.079	0.179	0.658	−0.430	0.271
ART experienced versus ART naïve	1.436	0.321	<0.001	0.807	2.065
Interaction between time and ART	−0.645	0.200	0.001	−1.036	−0.253
Males versus females	0.094	0.157	0.550	−0.214	0.401
Age (years)	0.028	0.007	<0.001	0.015	0.042
Body mass index at baseline	0.023	0.012	0.062	−0.001	2.028
Constant	1.839				

Note: standard error adjusted for 215 clusters in study ID at baseline; ART: antiretroviral therapy, significant *P* < 0.05.

**Table 3:** Comparison of lipid variables of ART naïve and ART experienced patients at baseline and follow-up.

	ART history						*P* ^*∗*^
	ART naïve (*n* = 23)		ART experienced (*n* = 148)		Total (*N* = 171)		
	Mean	SD	Mean	SD	Mean	SD	
Baseline TC/mmol/L	3.8	0.8	4.7	1.2	4.6	1.2	<0.001
Baseline HDL/mmol/L	1.0	0.2	1.3	0.4	1.2	0.4	<0.001
Baseline LDL/mmol/L	2.3	1.2	2.7	2.2	2.6	2.1	0.376
Baseline TC/HDL ratio	4.0	1.2	4.0	1.3	4.0	1.3	0.818

	ART history						*P* ^*∗∗*^
	ART naïve (*n* = 23)		ART experienced (*n* = 148)		Total (*N* = 171)		
	Mean	SD	Mean	SD	Mean	SD	

Follow-up TC/mmol/L	3.7	0.8	4.0	1.2	4.0	1.1	0.001
Follow-up HDL/mmol/L	1.4	0.4	1.3	0.5	1.3	0.5	0.002
Follow-up LDL/mmol/L	2.6	0.8	2.5	0.99	2.5	0.96	0.118
Follow-up TC/HDL ratio	2.9	1.04	3.5	2.4	3.4	2.3	0.841

Note: SD: standard deviation; level of significance is set at *P* < 0.05; ^*∗*^ *P* from *t*-test comparison of means; ^*∗∗*^ *P* from comparisons using generalised linear models (GLM) time *∗* group estimates. Mean values, for all 171 patients with complete baseline and follow-up data; TC: total cholesterol; LDL: low density lipoprotein cholesterol; HDL: high density lipoprotein cholesterol; ART: antiretroviral therapy.

**Table 4:** Comparison of patients with dyslipidemia at baseline and follow-up.

Baseline				
ART history				
Type of dyslipidemia	ART naïve (*n* = 23)	ART experienced (*n* = 148)	Total (*N* = 171)	*P* ^*∗*^
Elevated TC/*n* (%)	1 (4.4%)	55 (37.2%)	56 (32.8%)	0.020
Depressed HDL/*n* (%)	17 (73.9%)	77 (52.0%)	94 (55.0%)	0.050
Elevated LDL/*n* (%)	5 (21.7%)	34 (23.0%)	39 (22.8%)	0.896
Elevated TC/HDL ratio/*n* (%)	6 (26.1%)	35 (23.7%)	41 (24.0%)	0.799
Dyslipidemia/*n* (%)	19 (82.6%)	117 (79.1%)	136 (79.5%)	0.694

Nine-month follow-up				
ART history				
Type of dyslipidemia	ART naïve (*n* = 23)	ART experienced (*n* = 148)	Total (*N* = 171)	*P* ^*∗∗*^

Elevated TC/*n* (%)	0 (0%)	23 (15.9%)	23 (13.7%)	0.040
Depressed HDL/*n* (%)	9 (39.1%)	69 (46.6%)	78 (45.6%)	0.502
Elevated LDL/*n* (%)	2 (34.8%)	30 (20.3%)	38 (22.2%)	0.119
Elevated TC/HDL ratio/*n* (%)	2 (88.70%)	17 (11.6%)	19 (11.2%)	0.685
Dyslipidemia/*n* (%)	14 (60.9%)	96 (64.9%)	110 (64.3%)	0.710

Note: SD: Standard deviation; level of significance is set at *P* < 0.05; ^*∗*^ *P* from *t*-test comparison of means; ^*∗∗*^ *P* from McNemar chi square tests for paired data by time of visit; cutoff values according to NCEP guidelines [20]: elevated TC > 5.2 mmol/L, depressed HDL < 1.1 mmol/L, elevated LDL > 3.2 mmol/L, and elevated TC/HDL ratio > 4.5.

**Table 5:** Changes in frequency of patients with lipid derangements over 9 months.

ART history				
	ART naïve	ART experienced	Total	*P* ^*∗∗*^
Change in frequency of patients with elevated TC over nine months	−4.4%, *P* ^*∗*^ = 1.000	−23.5%, *P* ^*∗*^ = 0.281	−16.9%, *P* ^*∗*^ = 0.2288	0.002

Change in frequency of patients with depressed HDL over 9 months	−34.8%, *P* ^*∗*^ = 1.000	−5.4%, *P* ^*∗*^ < 0.001	−9.3%, *P* ^*∗*^ < 0.001	<0.001

Change in frequency of patients with elevated LDL over 9 months	+13.1%, *P* ^*∗*^ = 1.000	−2.6%, *P* ^*∗*^ < 0.001	−0.6%, *P* ^*∗*^ < 0.001	0.092

Change in frequency of patients with elevated TC/HDL ratio over 9 months	−17.4%, *P* ^*∗*^ = 0.688	−12.1%, *P* ^*∗*^ = 1.000	−12.7%, *P* ^*∗*^ = 0.7552	0.143

Change in frequency of patients with dyslipidemia over 9 months	−21.7%, *P* ^*∗*^ = 0.064	−14.2%, *P* ^*∗*^ < 0.001	−15.2%, *P* ^*∗*^ < 0.001	0.286

Note: SD: standard deviation; level of significance is set at *P* < 0.05; ^*∗*^ *P* from McNemar chi square tests for paired data by time of visit; ^*∗∗*^ *P* from McNemar chi square tests for paired data by ART groups; cutoff values according to NCEP guidelines [20]: elevated TC > 5.2 mmol/L, depressed HDL < 1.1 mmol/L, elevated LDL > 3.2 mmol/L, and elevated TC/HDL ratio > 4.5.
